# The Pomegranate Flower Water Extract Negatively Regulates Melanogenesis by Suppressing MITF Expression and Its Target Enzymes

**DOI:** 10.1111/jocd.70163

**Published:** 2025-04-15

**Authors:** Peng Shu, Nannan Xu, Nuermaimaiti Abudukelimu, Parida Aiziti, Deng Zang, Jiangyu Zhao, Yuan Wang

**Affiliations:** ^1^ HBN Research Institute and Biological Laboratory Shenzhen Hujia Technology Co., Ltd. Shenzhen China; ^2^ State Key Laboratory Basis of Xinjiang Indigenous Medicinal Plants Resource Utilization and Key Laboratory of Plant Resources and Chemistry of Arid Zone, Xinjiang Technical Institute of Physics and Chemistry Chinese Academy of Sciences Urumqi P.R. China; ^3^ University of Chinese Academy of Sciences Beijing P.R. China

**Keywords:** antimelanogenesis, cosmetic, melanin, pomegranate flower water extract, tyrosinase activity

## Abstract

**Background:**

The aesthetic issues caused by pigmentation are increasing people's demand for skin whitening. Considering its long‐term use, it is very important for searching safe and effective agents. Pomegranate flower, a kind of traditional Chinese medicine, has shown promising anti‐inflammatory, antioxidant, and antidiabetic properties, but its potential skin‐lightening effects have not been well explored.

**Aims:**

We investigated the effect of pomegranate flower water extract (PFE) on skin lightening and elucidated its underlying mechanisms.

**Methods:**

The radical scavenging capacity was measured by ABTS and DPPH assays, and the mechanism of lightening was detected by Western blot.

**Results:**

PFE could obviously inhibit tyrosinase activity, which its inhibition IC_50_ value was lower than the positive control, kojic acid. Meanwhile, the radical scavenging capacity was also better than vitamin C (VC). Then the synthesis ability of melanin was measured in B16F10 cells; we found that PFE, in its safe concentrations, could reduce the synthesis of melanin resulting from inhibiting TYR activities. The expression of the main melanogenesis enzymes TYR, TRP‐1, and TRP‐2 was sharply reduced. Interestingly, MITF, a transcription factor of TYR, was obviously decreased its expression when treated with PFE at 50, 100, and 150 μg/mL, which was even significantly lower than the downregulation by kojic acid.

**Conclusion:**

Pomegranate flower extract possessed a strong antimelanogenesis effect, which resulted from inhibiting the expression of MITF and its downstream target enzymes involved in melanin synthesis. These findings provide a strong scientific basis for the use of PFE as a safe and effective skin‐lightening ingredient in the cosmetic industry.

## 1 Introduction

Skin hyperpigmentation or its disorders give rise to appreciable impact on the quality of people's lives, carrying urgent and widespread demand for whitening skin. Skin lightening is a well‐documented and very old practice, which can be tracked back to the 16th century [1]. Lightening skin tone has been embedded in many cultures, including China, India, Korea, and Japan. Given that quite a lot of skin‐lightening products have emerged, which are not only the medical whitening agents applied to hyperpigmented lesions such as tretinoin, corticosteroids, and hydroquinone, but also include cosmeceuticals applied to lighten the skin like ascorbic acid, arbutin and kojic acid, and nicotinamide. Unfortunately, some agents are banned for use in cosmetic products for their side effects. So, it is very important to seek safe and effective whitening products. In Asia, people pay more attention to the traditional herbs in uniformizing skin tone since the culture and aesthetic manner of Asians and the long history of using herbal medicine [2].

In order to screen a safe and effective herb plant in lightening skin, an overview of the skin pigmentation pathway should be understood. Skin color is determined by a mixture of four biochromes: reduced hemoglobin (blue color), oxygenated hemoglobin (red color), melanin (orange‐black color), and carotenoids (yellow color). Significantly, melanin is the vital determinant of the skin, hair, and eye color. Melanin is the production of melanocyte, which can survive considerable genotoxic stress and absorb ultraviolet radiation. Melanocytes in the skin are surrounded by keratinocytes, in which one melanocyte is surrounded by almost 36 keratinocytes [3]. In this way, melanosomes, which reside in melanocyte and produce melanin, can be transferred into their neighboring keratinocytes [4]. Interestingly, although all races have the same numbers of melanocytes, the number and distribution of the melanosomes produce determine the skin color [5]. The key enzyme in melanin synthesis is tyrosinase (TYR), its transcription is controlled by the key regulator of melanogenesis, termed microphthalmia‐associated transcription factor (MITF). MITF is a very vital transcription factor in melanogenesis and acts as a conscription activator to regulate the expression of TYR, TYRP1 (tyrosinase‐related protein 1), and other genes, to promote melanogenesis [6]. In mammals, melanomas produce two types of melanin: red or yellow phenomenal and brown or black melanin. TYR, acting as the rate‐limiting protein, catalyzes the direct or indirect oxidation of L‐tyrosine to form dopaquinone [7]. Two other tyrosinase‐related enzymes, the tyrosinase‐related proteins‐2 or dopachrome tautomerase (TRP‐2 or Tyrp2 or DCT) and the tyrosinase‐related proteins‐1 (TRP‐1 or Tyrp1), catalyze the synthesis of melanin. DCT catalyzes the conversion of dopaquinone to 5,6‐dihydroxyindole‐2‐carboxylic acid (DHICA); then, TYRP1 catalyzes the oxidation of DHICA to form eumelanin. However, in the absence of the enzyme DCT, the dopaquinone can also continually decarboxylate and polymerize to form 5,5‐dihydroxyindole (DHI) melanin [8]. In pheomelanogenesis, L‐DOPAquinone reacts to produce several cysteinyl‐DOPA (CD) isomers like 2‐S‐cysteinyl‐DOPA (2SCD), 5‐S‐cysteinyl‐DOPA (5SCD), 2,5‐S, S′‐cysteinyl‐DOPA (DiCD), or ortho‐quinonimine (ortho‐QI) in the presence of L‐cysteine or glutathione. Then, further oxidation of these thiol adducts and their polymerization leads to the assembly of the yellow‐red pheomelanin via benzothiazine intermediates [8] (Draft). By now, most skin‐lightening agents play the whitening function through inhibiting the activity of tyrosinase.

**Figure:**
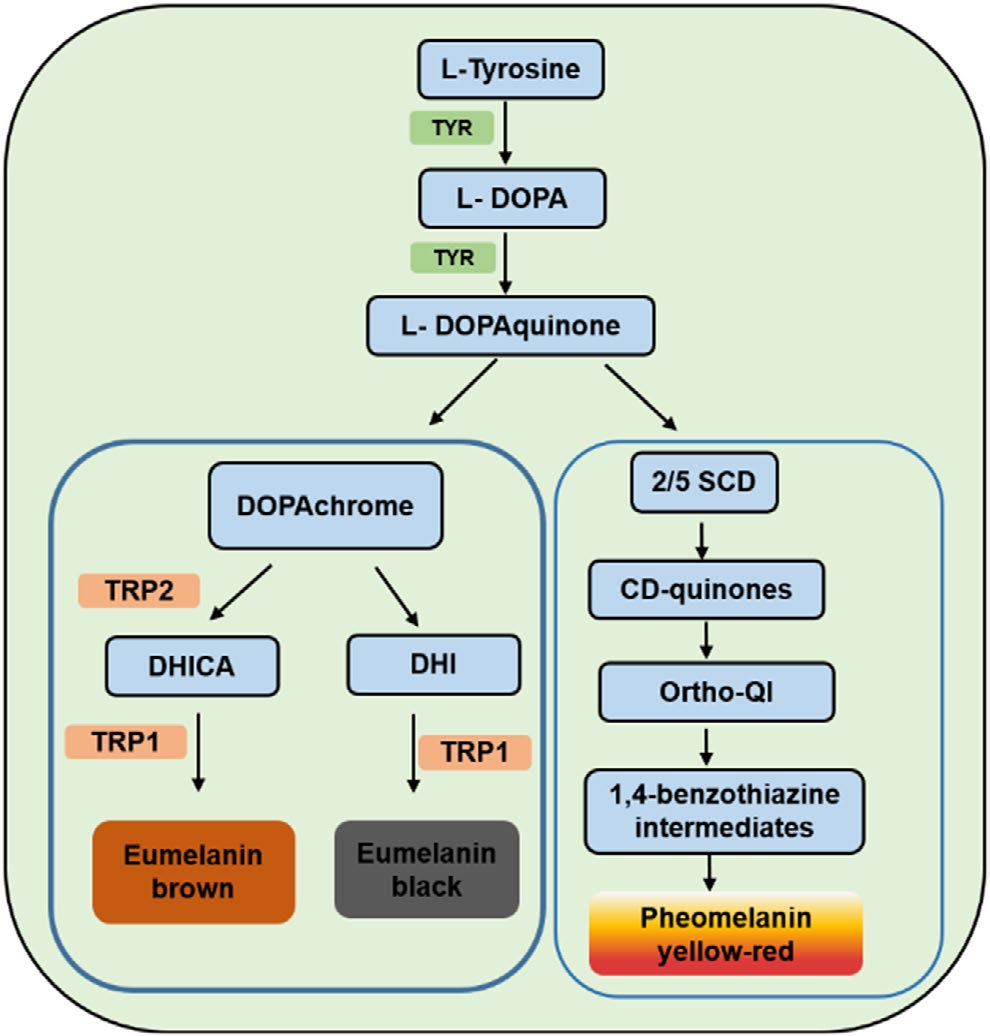
Draft. Biosynthetic pathways of eumelanin and pheomelanin.

The pomegranate flower (PGF), an ancient herb resulting from deciduous shrubs or small *Pomegranates*, which are members of the genus *Pomegranate*, causes a series of scientists' research on its medicinal function. In traditional Chinese medicine, the PGF is applied to inflammation or bleeding. But in Uyghur medicine, the PGF is used for treating cataracts, nausea, and neurasthenia. Meanwhile, it is also used to treat diabetes in India [9]. In addition to various applications in medicine, the active ingredients in PGF have been analyzed, including tannins, phenols, sugars, trace elements, pigments, and polyphenols. The PGF has good pharmacological activities, including anti‐inflammation, hypolipidemic, and antidiabetes [10], inhibiting cancer proliferation [11], antibacterial activities, and antioxidation [12], and anthelmintic activity for its pelletriene alkaloid [13]. Significantly, the PGF extract also possesses tyrosinase inhibitory activity in vitro. However, it is unclear whether the PGF has the ability to reduce melanin production at the skin cell level and its regulation mechanism on inhibiting the synthesis of melanin.

Therefore, we tried to obtain the pomegranate flower water extract (PFE) and to study whether PFE could alleviate the synthesis of melanin in B16F10 cells, and further investigated the reason. Interestingly, it was confirmed by cell and some experiments in vitro that PFE could inhibit melanogenesis. Its mechanism is relevant to the fact that PFE inhibited the expression of MITF, which downregulated the expression of the key enzyme TYR and the associated proteins TRP‐1 and TRP‐2. This is great significant for searching for safe and effective lightening skin additives and improving the industrial value of pomegranate flower.

## 2 Materials and Methods

### 2.1 Materials and Reagents

L‐3,4‐dihydroxyphenylalanine (L‐DOPA), DPPH, and mushroom tyrosinase were purchased from Sigma‐Aldrich; their catalogs are D9628, D9132, and T3824, respectively. Forskolin was purchased from MCE (MedChemExpress, HY‐15371). Kojic acid was purchased from Solarbio (K8070). Primary antibodies for TYR, TRP‐1, and TRP‐2 were obtained from Santa Cruz Biotechnology (A1323, A2423, L0513). Furthermore, the antibody for MITF was purchased from Cell Signaling Technology (97800S). β‐Actin and antibodies designed to target primary antibodies were obtained from BOSTER Biological Technology (Wuhan, China). The reference compounds gallic acid and ellagic acid were supplied by the National Institutes for Food and Drug Control (NIFDC; Beijing, China). The reference compound corilagin was purchased from Shanghai PureOne BioTech Co. Ltd. (Hong Kong, China). Methanol, acetonitrile, and formic acid were purchased from Thermo Fisher Scientific (Fair Lawn, NJ, USA). Deionization water was obtained from Watson's (Hong Kong, China).

### 2.2 PFE Preparation

Crushed 10 g of pomegranate flowers, then added 250 mL of deionized water, and fluxed for 2 h. Filtered and separated the drug residue, and concentrated the extracted solution through a rotary evaporator at 50°C to obtain a concentrated solution; freeze‐dried and crushed the concentrated solution to obtain the extract sample and purified [14]. Pomegranate flowers were purchased from Hotan, Xinjiang province, which were identified by XinJiang Institute of Ecology and Geography Chinese Academy of Sciences.

### 2.3 Radical Scavenging Activity Assay

#### 2.3.1 DPPH Assay

The antioxidant effect of PFE was assessed based on its scavenging activity on 1,1‐diphenyl‐2‐picrylhydrazyl (DPPH) free radicals using the modified DPPH assay [15]. Prepared 2 mmol/L DPPH stock solution and saved it at −20°C under dark condition. Ensured the absorbance of diluted DPPH solution is about 0.70 ± 0.02 when detected at 515 nm in each experiment. Incubated 100 μL of PFE solution and 100 μL diluted DPPH solution for 30 min and then detected the absorbance at 515 nm. Vitamin C acted as a positive control. Inhibition percentage (%) = [1 − (OD_samples_ − OD_control_)/OD_blank_] × 100%.

#### 2.3.2 ABTS Assay

The modified version of the method was used for the 2,2′‐azino‐bis (3‐ethylbenzothiazoline‐6‐sulfonic acid) (ABTS) free radical scavenging assay [16]. A 10 mmol/L ABTS stock solution was prepared and saved in dark condition. The ABTS working solution was diluted when the absorbance was about 0.70 ± 0.02 at 734 nm wavelength. Then, 16 μL PFE solution was mixed with 184 μL ABTS solution and incubated for 5 min at room temperature, then the absorbance was measured at 734 nm. Vitamin C acted as a positive control. Inhibition percentage (%) = [1 − (OD_samples_ − OD_control_)/OD_blank_] × 100%.

### 2.4 Tyrosinase Inhibitory Assay

The mushroom tyrosinase inhibition assay was conducted using this modified method [17]. Added 40 μL PBS (10 mM, pH 6.0), PFE solution, and 25 U/mL of mushroom tyrosinase and incubated for 10 min. Then L‐DOPA (10 mM) was added and incubated for 10–20 min at 37°C. The absorbance at 490 nm was measured by using Spectra Max M5 (Molecular Devices Company, San Jose, CA, USA). Kojic acid (KA) was employed as a positive control. Tyrosinase inhibition (%) = (1 − (OD_samples_ − OD_blank_)/(OD_control_ − OD_blank_)) × 100%.

### 2.5 Cell Culture and Cell Viability Assay

The murine melanoma cells B16F10 were purchased from BeNa Culture Collection (BNCC100309). The cells were cultured with 1640‐medium containing 10% fetal bovine serum (BeNa Culture Collection) and 1% penicillin/streptomycin. CCK‐8 assay (Beyotime, C0038) was used to evaluate the cytotoxic activity of the tested sample. Eight thousand cells were seeded in 96‐well plates per well and incubated overnight. The fresh medium containing PFE was added to each well and incubated for about 24 h. Then the medium was cleared and 100 μL CCK‐8 working solution was added for 1–2 h. The absorbance at 450 nm was measured and the cell proliferation rate was calculated.

### 2.6 Measurement of Cellular Melanin Contents

The melanin content of treated B16F10 cells was measured [18]. Seeded B16F10 cells at about 0.5 × 10^5^ cells/well into a 6‐well plate and incubated overnight. Changed the fresh medium contained 700 μM kojic acid (positive control) and PFE, respectively. Then added 4 μM forskolin after 30 min, incubated for 24 h, then continue added 4 μM forskolin and incubated for 48 h. Harvested the cells with RIPA, centrifuged at 12 000 rpm at 4°C for 22 min. Pipetted 3 μL supernatants and measured the protein concentration. Removed the supernatants and added 200 μL NaOH (1 M) lysis incubating for 60 min at 80°C. Next, measured the absorbance at 405 nm. Finally, the melanin content in cells was calculated. Each experiment was performed three times.

### 2.7 Intracellular Tyrosinase Activity Assay

The method was slightly modified, which referred to this reference [18]. 1 × 10^5^ cells/well were seeded into a 6‐well plate and incubated overnight. Changed fresh medium contained 700 μM kojic acid (positive control) and PFE, respectively. Then added 4 μM forskolin after 30 min, incubated for 24 h, then continue added 4 μM forskolin, incubated for 24 h. Lysed the cells with PBS contained 1%TritonX‐100 and sodium deoxycholate. Centrifuged at 12 000 rpm for 22 min at 4°C. Measured the protein concentration using the BCA kit. Then pipetted 60 μL supernatant and added 10 μL L‐DOPA. Incubated for 10–20 min at 37°C and measured its absorbance at 490 nm.

### 2.8 Western Blot Assay

Lysate the cells by RIPA lysis buffer containing protein phosphatase inhibitor and 1 mM phenylmethylsulphonyl fluoride (PMSF). The protein concentration was measured by BAC kit. Added 30 μg proteins per lane and separated the proteins by SDS‐PAGE gels. Then transferred to PVDF membranes for 180 min at 400 mA. Blocked with blocking buffer including 5% skim milk for 1 h at room temperature; then the corresponding antibodies were incubated overnight at 4°C, washed with tris buffered saline with 0.1% Tween‐20 (TBST) three times, each time for 10 min, and incubated the secondary antibody for 1 h at room temperature, washed with TBST three times, each time for10 min. Chemiluminescence was imaged by ChemiDocMP Imaging System (Bio‐Rad Laboratories, Hercules, CA, USA), and the protein content was measured by Image J software.

### 2.9 Qualitative Analysis of PFE

Chromatographic separation was performed using an Ultimate 3000 UHPLC system (UHPLC–MS/MS, Thermo Fisher Co., Germany). The chromatography was performed on a Waters ACQUITY UPLC BEH Shield RP18 (2.1 × 100 mm, 1.7 μm; Waters, Wexford, Ireland) column. The mobile phase consisted of 0.1% aqueous formic acid (A) and acetonitrile (B); the flow rate was 0.2 mL/min, the column temperature was 40°C, and the injection volume was 2 μL. The gradient elution was as follows: 0–4 min, 2% B; 4–30 min, 2%–41% B; and 30–50 min, 41%–95% B.

High‐resolution MS data were recorded on a Q‐Exactive hybrid quadrupole‐Orbitrap mass spectrometer equipped with a heated ESI source (Thermo Fisher Scientific, Bremen, Germany) operating in positive and negative ion modes. The heated ESI (HESI) parameters were set as follows: capillary temperature, 350°C; sheath gas pressure, 40 arb; aux gas pressure, 10 arb; voltage, 3.5 kV in positive mode and 2.8 kV in negative mode. The scanning modes were Full MS/dd‐MS^2^. The scanning range was m/z 100–1500 at a resolution of 70 000 for full scan and 17 500 for MS/MS. The MS data were viewed and processed by Xcalibur 4.6 (Thermo Fisher Scientific, Waltham, MA, USA).

### 2.10 Statistical Analysis

The results are expressed as mean ± standard deviation (SD). If *p* < 0.05, the difference was considered significant.

## 3 Results

### 3.1 Identification of the Main Components of PFE

In order to better study the bioactive of PFE, the main components of PFE were identified by UHPLC–MS/MS in negative ionization modes (Figure S1). Based on the full MS/dd MS^2^ scan mode, the exact MS information was obtained, and the main chemical components were further inferred. Twenty‐one main compounds were identified from the PFE according to the mass data and the previous literature [19], including ellagic acid, galloyl hexoside, punicalin, digalloyl hexoside, corilagin, brevifolin, trigalloyl hexoside, ellagic acid hexoside, and the like (Table S1).

### 3.2 Tyrosinase Inhibition and Antioxidation Potential In Vitro

**FIGURE 1:**
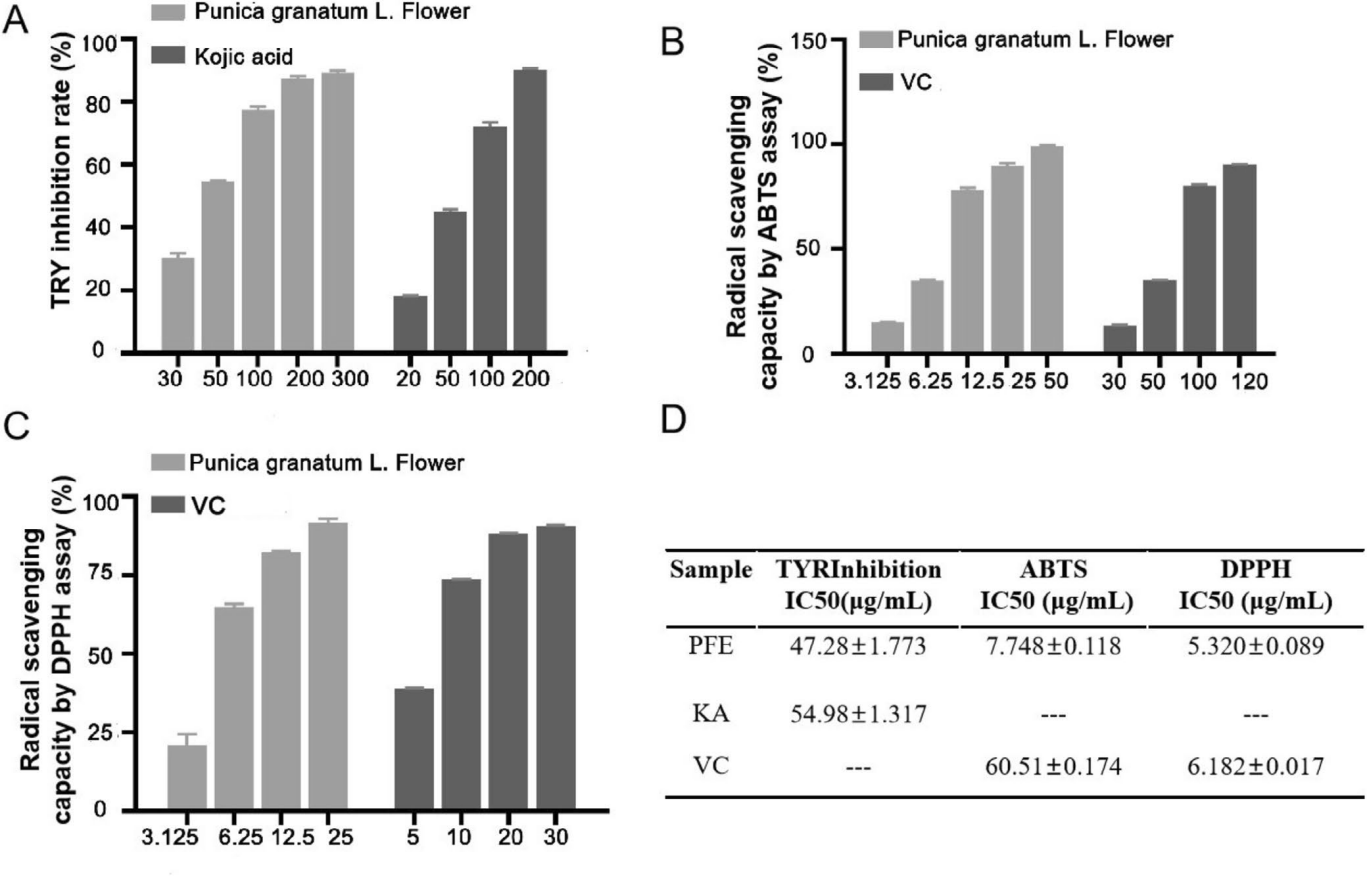
The PFE has significant skin‐lightening potential. (A) The PFE strongly inhibited mushroom tyrosinase in vitro. Kojic acid as a positive control. (B) Radical scavenging capacity by ABTS assay. VC acts as a positive control. (C) Radical scavenging capacity by DPPH assay. VC acts as a positive control. (D) The IC50 value of TYR inhibition assay, ABTS assay, and DPPH assay. Results were presented as the mean ± SD (*n* = 3).

We investigated the inhibition activity of cumulative concentrations of PFE (30, 50, 100, 200, and 300 μg/mL) on mushroom tyrosinase in vitro. Kojic acid (KA) is a hydrophilic fungal derivative from aspergillus bacteria that is commonly used in cosmetic preparations for whitening skin [5]. Here, kojic acid serves as the positive control. When 50 μg/mL PFE was added, the tyrosinase inhibition rate was about 55.09% ± 0.4453%, better than that of the 50 μg/mL kojic acid, which had a tyrosinase inhibition rate of about 45.14% ± 0.6152% (Figure 1A). It was determined that the IC_50_ of TYR inhibition is about 47.28 ± 1.773 μg/mL, which is superior to the 54.98 ± 1.317 μg/mL of kojic acid (Figure 1D).

Ultraviolet (UVB) radiation‐induced ROS trigger melanogenesis. The agents with ROS‐scavenging ability prevent oxidative melanogenesis reactions to promote skin lightening, such as vitamin C (VC) [20]. Therefore, we measured the radical scavenging capacity by the ABTS assay. When 3.125 μg/mL of PFE was added, the radical scavenging capacity was better than 30 μg/mL of VC; even 12.5 μg/mL of PFE was superior to 50 μg/mL of VC (Figure 1B). Significantly, the IC_50_ of PFE for radical scavenging capacity by ABTS is about 7.748 ± 0.118 μg/mL. However, the positive control of VC is 60.51 ± 0.174 μg/mL (Figure 1D). Meanwhile, the IC_50_ of PFE for radical scavenging capacity by DPPH is about 5.320 ± 0.089 μg/mL, while the VC is 6.182 ± 0.017 μg/mL (Figure 1C,D). In summary, PFE has significant skin‐lightening potential.

### 3.3 Effect of PFE on B16F10 Cell Viability

**FIGURE 2:**
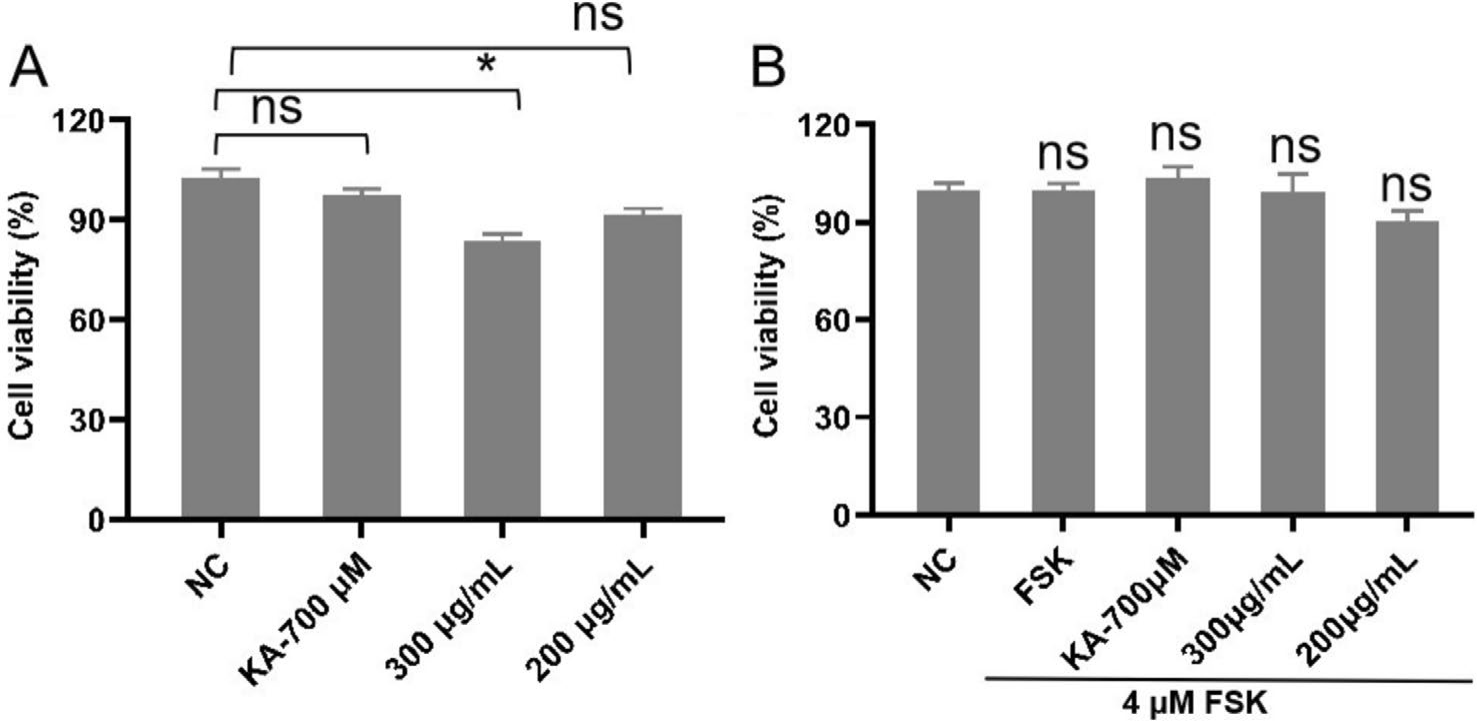
Cell viability. (A) Cell proliferation of accumulated concentration of PFE on B16F10 cells. Kojic acid (KA) as positive control, the working concentration is 700 μM. (B) Cell proliferation of accumulated concentration of PFE on B16F10 cells when stimulated with forskolin. The PFE concentrations were 200 and 300 μg/mL, respectively. NC, normal cells. Results were presented as the mean ± SD (*n* = 3; *p < 0.05 compared with the NC group).

B16F10 cells were initially seeded in microplates followed by different concentrations of PFE. Treating B16F10 cells with PFE (0–200 μg/mL) did not affect the viability of B16F10 cells (Figure 2A). However, when treating B16F10 cells with 300 μg/mL of PFE alone, the cell viability was less than 90%, which demonstrated that this dose was toxic to B16F10 cells. But when adding 4 μM forskolin (FSK) [17] together with 300 μg/mL of PFE to B16F10 cells, there was no toxicity. This may result in the synergistic effect of forskolin.

### 3.4 Inhibition Effect of PFE on Melanin Content and Tyrosinase Activity

**FIGURE 3:**
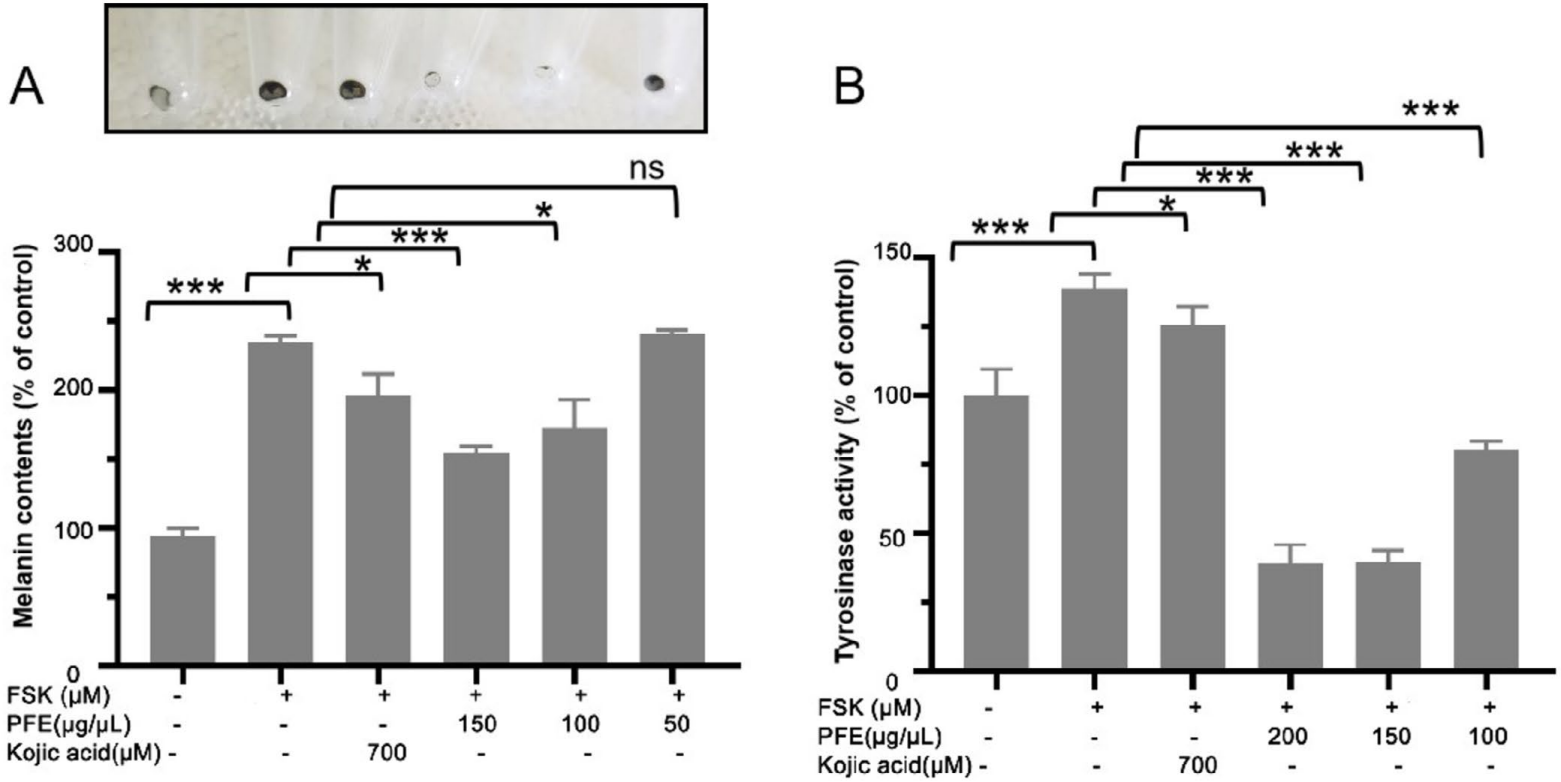
PFE strongly inhibited tyrosinase activity and reduced the synthesis of melanin. (A) Mensuration of melanin content in B16F10 cells. (B) Mensuration of tyrosinase activity in B16F10 when treated with PFE. Results were presented as the mean ± SD (*n* = 3; *p < 0.05, ***p < 0.001 compared with the forskolin‐stimulated group).

The efficacy of obtained PFE as inhibitors of melanin synthesis was further investigated in vitro by using B16F10 murine melanoma cells. The cell line can synthesize and release plentiful melanin and is an experimental model for studying melanogenesis inhibitors. For this study, three concentrations of PFE were chosen. As shown in Figure 3A, 100 and 150 μg/mL of PFE decreased the synthesis of melanin stimulated with FSK. Furthermore, in a mushroom tyrosinase inhibitory assay, 100 μg/mL of PFE still showed a good tyrosinase inhibition in B16F10 cells although the activity of tyrosinase inhibition was lower than that treated with 150 and 200 μg/mL of PFE (Figure 3B). Even 200 and 150 μg/mL of PFE could significantly decrease tyrosinase activity, which the tyrosinase activity is both about 39% to that of control. Considering the above study, the PFE could reduce the synthesis of melanin by inhibiting tyrosinase activity.

### 3.5 Effect of PFE on FSK‐Induced Melanin Synthesis Pathway

**FIGURE 4:**
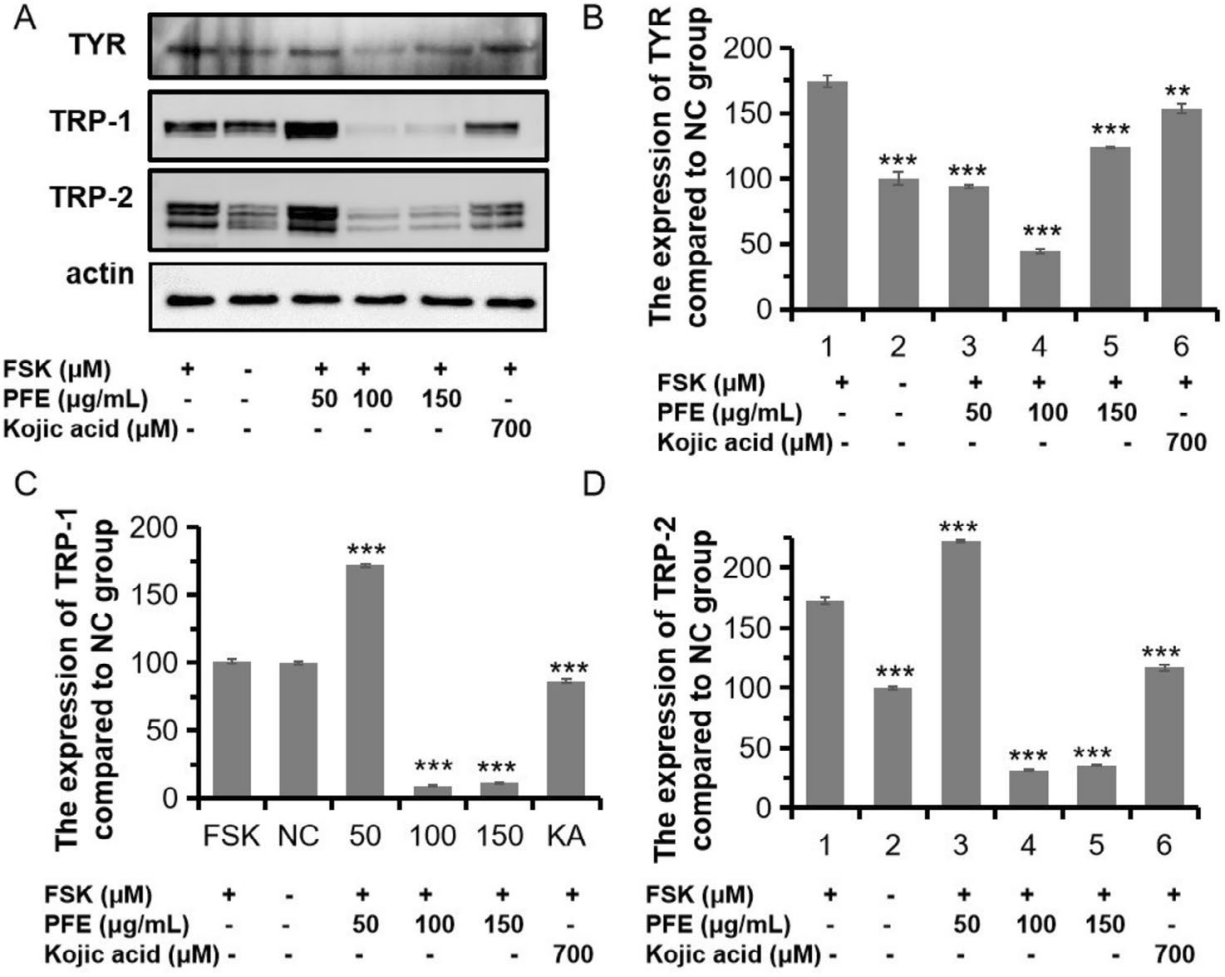
PFE strongly inhibited tyrosinase activity and TYR‐associated proteins. (A) The proteins' expression of main enzymes of melanogenesis. The primary antibodies were TYR, TRP‐1, and TRP‐2. (B–D) The grayscale value of the expression of TYR, TRP‐1, and TRP‐2 when compared to the forskolin‐stimulated group. Results were presented as the mean ± SD (*n* = 3; **p < 0.01, ***p < 0.001).

Given that the significant inhibition of melanin synthesis of PFE, we investigated the mechanism of PFE on the TYR, the limiting enzyme for melanin synthesis. As shown in Figure 4A,B, the PFE could sharply decrease the expression of TYR to 93.92%, 43.92%, and 123.95% compared to that of the FSK‐induced group when treated with 50, 100, and 150 μg/mL of PFE. Those three doses both preferred to that of KA treated (153.65%). Meanwhile, two similar proteins with tyrosinase, occupied 40% homologous amid acids, are tyrosinase‐related protein‐1/2 (TRP‐1/2). They are all localized on the membrane of melanosomes. Although the function of these two enzymes is unclear, the TRP‐1 may possibly act on activation and stabilization of TYR, the synthesis of melanosome, and determine the ratio of eumelanin/pheomelanin. The TRP‐2 plays a role in a dopachrome tautomerase like TYR [21]. Therefore, we measured the expression of the two proteins. As expected, the expression of TRP‐1 and TRP‐2 was significantly reduced when compared to the FSK‐induced group. The PFE added with 100 and 150 μg/mL could significantly decrease the expression of TRP‐1 and TRP‐2 than the FSK‐induced group (Figure 4). However, 150 μg/mL of PFE could moderately increase the expression of TRP‐1 or TRP‐2, which should be studied in later research. In conclusion, PFE decreased the synthesis of melanin by inhibiting the expression of main enzymes TYR, TRP‐1, and TRP‐2.

### 3.6 Effect of PFE on MITF

**FIGURE 5:**
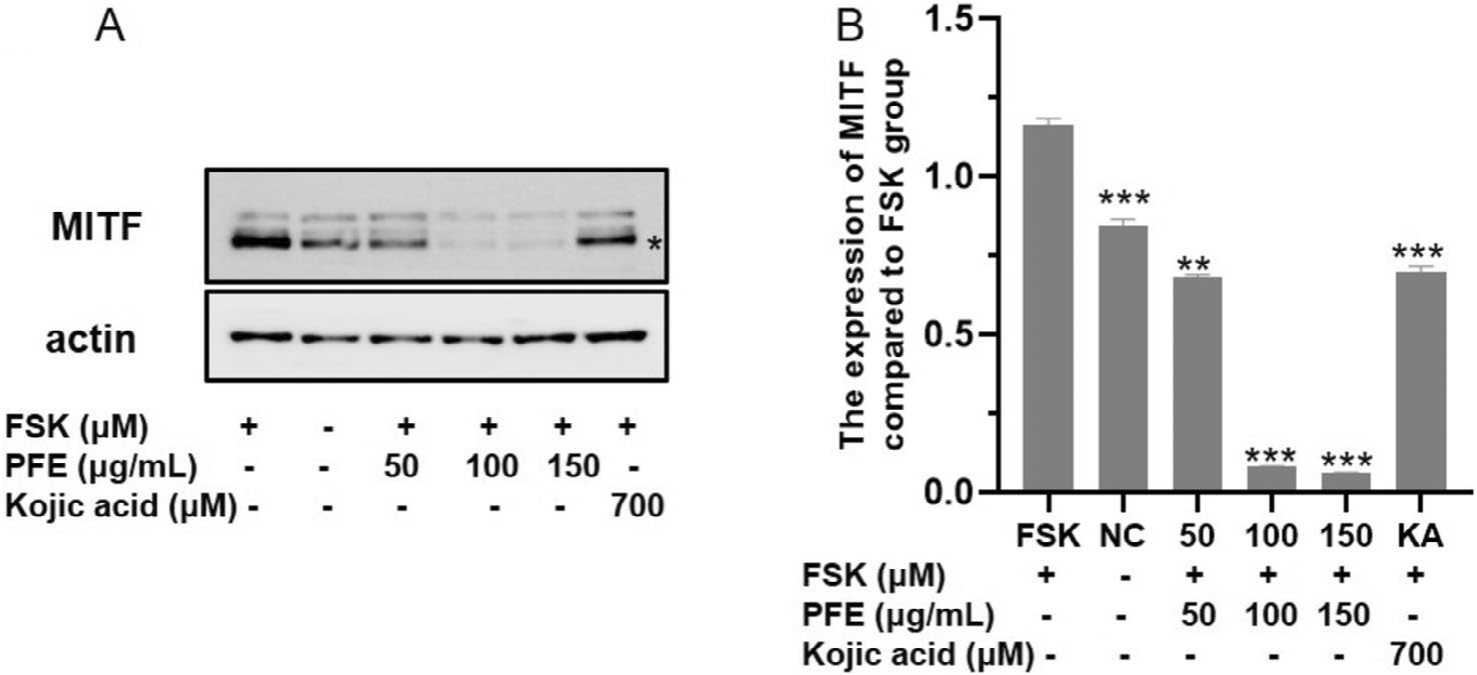
PFE inhibited the expression of MITF. (A) The proteins' expression of MITF. (B) The grayscale value of the expression of MITF when compared to the forskolin‐stimulated group. Results were presented as the mean ± SD (*n* = 3; **p < 0.01, ***p < 0.001).

MITF, the transcription factor, plays a pivotal role in the expression of numerous differentiation factors and pigment enzymes [3], such as TYR. Therefore, the expression of MITF was examined. As shown in Figure 5, the content of MITF is seriously decreased in the PFE group treated with 50, 100, and 150 μg/mL compared to the FSK‐induced group, and it is even obviously reduced compared to the KA‐treated group. Considering the result, the appropriate PFE inhibited the synthesis of melanin through downregulating the expression of MITF, which resulted in reducing the expression of numerous melanogenesis‐related enzymes: TYR, TRP‐1, and TRP‐2.

## 4 Discussion

The pomegranate flowers play numerous functions in anti‐inflammatory, antidiabetes, antilipid, and antioxidation. From a previous study, the main components of pomegranate flower ethanol extract were galloyl‐HHDP‐glucoside, granatin A, Digalloyl‐glucoside, tri‐O‐galloyl‐glucoside, granatin B, ellagic acid, ellagic acid derivative, ellagic acid rhamnoside, quercetin, quercetin‐O‐glucoside, kaempferol, and luteolin [22]. As we know, some components in here were involved in melanogenesis and antioxidant activities. Ellagic acid (EA), as a phenolic compound, has been confirmed as a tyrosinase inhibitor, which resulted from it being oxidized by TYR and producing the O‐quinones [23]. Interestingly, ellagic acid also possesses strong antioxidant activities in which the value of IC_50_ in scavenging ABTS and DPPH are 4.59 ± 0.07 and 10.54 ± 0.07 μg/mL, respectively [24]. However, in our study, the scavenging ABTS and DPPH of PFE were 7.748 ± 0.118 μg/mL, which is higher than that of ellagic acid and 5.320 ± 0.089 μg/mL, lower than that of ellagic acid. Kaempferol also has TYR activities in which the IC_50_ value is 171.4 ± 0.9 μM [25]. Quercetin, as a main component in pomegranate flower ethanol extract, decreases the intracellular tyrosinase activity [26] against melanogenesis by mouse melanoma cells and improves the antioxidant capacity by regulating MAPK, NRFB, AMPK, and other signaling pathways induced by ROS [27]. Besides those, galloyl‐HHDP‐glucoside [28], luteolin [29], and kaempferol [30] also have antioxidant activities in previous studies. However, the main components of PFE here were also plentiful, such as ellagic acid, galloyl hexoside, punicalin, digalloyl hexoside, corilagin, brevifolin, trigalloyl hexoside, and ellagic acid hexoside. Previous studies reported that corilagin extracted from longan seed also shows tyrosinase inhibition activities and antioxidation activities in which the IC50 value of tyrosinase inhibitory activity was 1231 mM, 9.23 ± 0.25 μM for DPPH assay, and 42.93 ± 2.39 μM for ABTS assay, respectively [31]. In addition, punicalin [32], digalloyl hexoside [33], brevifolincarboxylic acid [34, 35], trigalloyl hexoside [36], and brevifolin [37] also possesses antioxidant activities. In summary, there are some main components in PFE that supported the antioxidant activities and tyrosinase inhibitory activities. However, which compound was the main support for the antioxidant activities and/or tyrosinase inhibitory activities should be further isolated and identified.

In recent publication, Gurale et al. also found that the flowers of *Punica granatum* methanolic extract (PFM) were performed on inhibiting tyrosinase activity in vitro enzyme activity experiment in which the IC50 value is 76.01 μg/mL and owned radical scavenging activity by DPPH method [38], which the IC50 value is 49.81 μg/mL. Similarly, we also found that PFE possessed the function on inhibiting tyrosinase activity in vitro in which the IC50 value was 47.28 ± 1.773 μg/mL and owned strong radical scavenging activity by DPPH method in which the IC50 value was 5.320 ± 0.089 μg/mL. This result demonstrated that the PFE possesses more superior tyrosinase inhibition activity and radical scavenging activity by DPPH method than flowers of *P. granatum* methanolic extract (PFM). In Gurale's experiment, the PFM also had lipid peroxidation inhibitory activity using rat liver homogenate and hydroxyl radical scavenging activity by deoxyribose method. But we investigated that the PFE occupied the radical scavenging capacity by using ABTS method. Moreover, the tyrosinase activity of PFE was surveyed in B16F10 cells, and the melanin content was reduced when treated with PFE in our study. Furthermore, the mechanism of antimelanogenesis of PFE was studied here. Otherwise, we found that when added 50 or 100 μg/mL of PFE, it could sharply decrease the expression of TYR compared to that of only FSK‐induced group, in which it presented in a dose‐dependent manner (Figure 4A,B). This result demonstrated that 100 μg/mL of PFE occupied a more strong inhibited function of the expression of TYR. Meanwhile, the expression of TYR was moderately lower (123.95%) than that of only FSK‐induced group when treated with 150 μg/mL of PFE in FSK‐induced group. The higher expression of TYR when added 150 μg/mL of PFE to B16F10 cells might result from another signal pathway that was activated in which improved the expression of TYR proteins.

As we know, melanogenesis is affected by multiple factors, namely, nonspecific internal pathways (e.g., inflammatory, hormones factors) [39], the external environment (e.g., chemical drugs, solar radiation), epigenetic regulation (e.g., histone modification, DNA methylation, chromatin remodeling and noncoding RNA) [40], paracrine factors from fibroblasts and keratinocytes, such as ET‐1, SCF, and alpha‐melanocyte stimulating hormone (α‐MSH) [41], cellular autograph of melanocyte, keratinocyte, and fibroblast in skin [42], and the specific internal signaling pathways, which include mitogen‐activated protein kinase (MAPK), cyclic adenosine monophosphate (cAMP)/protein kinase A (PKA), phosphatidylinositol‐3‐kinase (PI3K)/Akt, Wnt/β‐catenin, and SCF/c‐Kit signaling pathways [43, 44]. Most of them can monitor the activity and the expression of MITF. However, in our study, we only measured the key melanin synthesis pathway and checked the expression of the key enzyme TYR. The TYR‐related enzymes TRP‐1 and TRP‐2 were also checked, along with the expression of their transcription factor MITF. Therefore, it is not clear which signaling pathways above regulate MITF and whether PFE plays the skin‐lightening role through another mechanism. Furthermore, all the above questions should be studied in depth.

## 5 Conclusion

In summary, this study demonstrated that the PFE could significantly reduce the synthesis of melanin by decreasing the expression of MITF in B16F10 cells, which resulted in inhibiting the expression of TYR, TRP‐1, and TRP‐2. These data confirmed that the pomegranate flowers are a potent antimelanogenic agent and can act as a depigmenting agent in the preparation of topical skin‐lightening agents.

## Author Contributions

Peng Shu mainly engaged in the whitening mechanism. Nannan Xu wrote and edited the manuscript. Nuermaimaiti Abudukelimu participated in the extraction and separation of PFE. Parida Aiziti completed the experiments of DPPH and ABTS. Deng Zang completed the experiment of TYR inhibition in vitro. Jiangyu Zhao modified the draft. Yuan Wang was responsible for the idea, review, and editing. All authors have read and approved the submitted version.

## Conflicts of Interest

The authors declare no conflicts of interest.

## Supporting information


**Figure S1.** The main components of PFE were identified by UHPLC–MS/MS.

**Table S1.** Characterization of the main compounds in PFE by UHPLC–MS/MS (negative ionization modes).

## Data Availability

All data generated or analyzed in this study are included in this published article.
