# Supplementary figures and images for: The Pomegranate Flower Water Extract Negatively Regulates Melanogenesis by Suppressing MITF Expression and Its Target Enzymes
Source: J Cosmet Dermatol. 2025 Apr 15;24(4):e70163. doi: 10.1111/jocd.70163 (PMC11998895; doi:10.1111/jocd.70163)

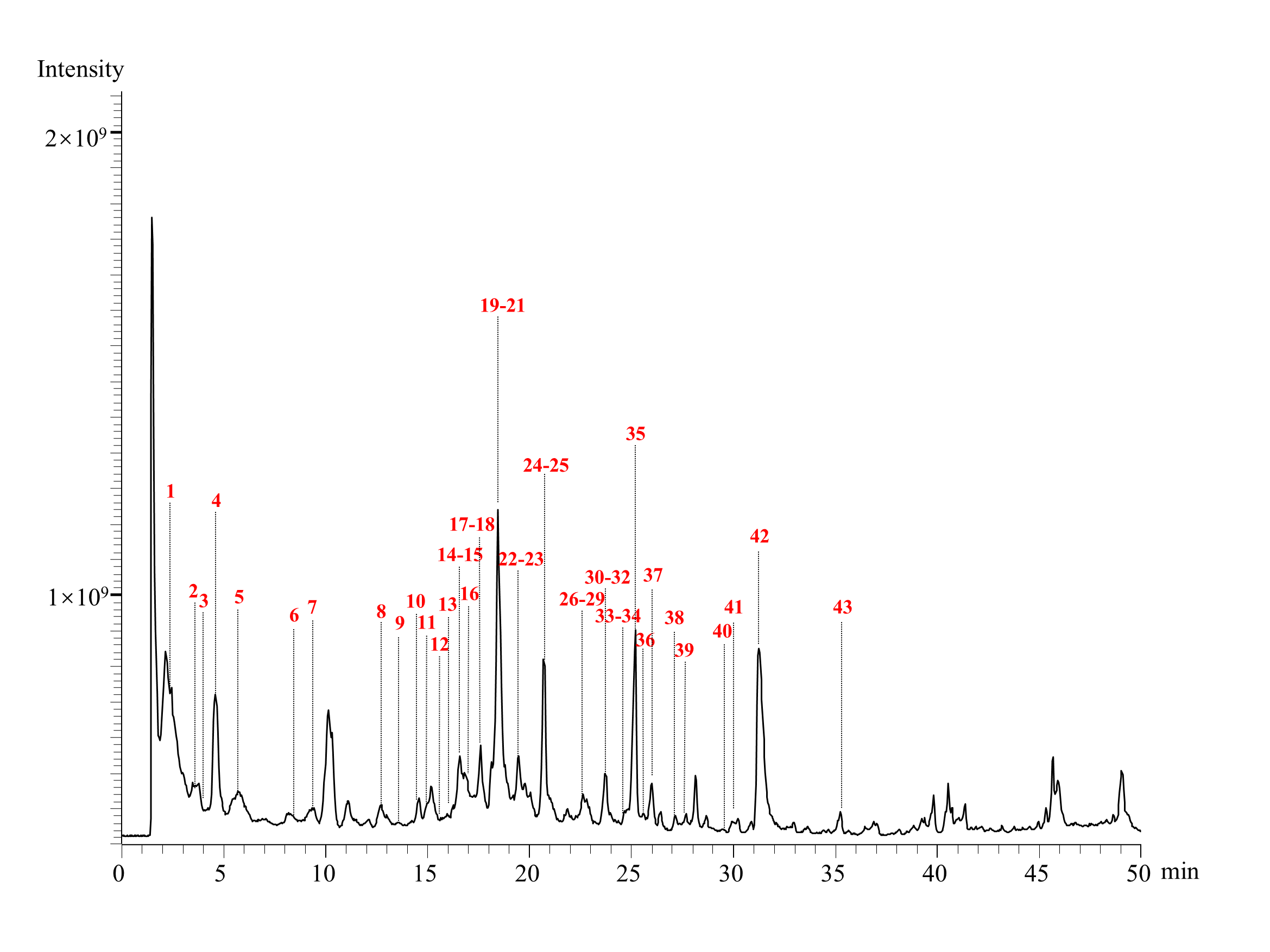

Supplement: Supplementary file 1 — Figure S1. The main components of PFE were identified by UHPLC–MS/MS. [file JOCD-24-e70163-s002.tif]
